# Monthly Intramuscular Neridronate for the Treatment of Postmenopausal Osteoporosis: Results of a 6-Year Prospective Italian Study

**DOI:** 10.1155/2019/9802827

**Published:** 2019-02-06

**Authors:** L. Guiducci, C. Vassalle, P. Parchi, S. Maffei

**Affiliations:** ^1^Institute of Clinical Physiology, CNR National Research Council, Pisa, Italy; ^2^CNR-Regione Toscana G Monasterio Foundation, Pisa, Italy; ^3^1st Orthopedic Division, Department of Translational Research and New Technology in Medicine and Surgery, University of Pisa, Pisa, Italy

## Abstract

**Purpose:**

Oral bisphosphonates (BPs) are the most commonly used medications for osteoporosis (OP), but their poor gastrointestinal (GI) absorption and tolerance hamper compliance. Intramuscular (IM) neridronate (NE), an amino-BP, is an easy-to-administer, effective, and safe alternative to oral BPs. We assessed the 6-year effects of monthly IM NE on bone mineral density (BMD) and bone turnover biomarkers (BMs) in postmenopausal OP.

**Methods:**

This single-center, prospective study enrolled postmenopausal osteoporotic outpatients with gastric intolerance to BPs (based on Tuscany Region's law GRT n. 836 20/10/2008). They received 25 mg IM NE once a month (with vitamin D and calcium if necessary) for 6 years. BMD was evaluated at lumbar spine (L1-L4), femoral neck (FN), and total femur (TF) at baseline (BL) and every 12 months afterwards. At BL, month 3, and every 12 months after BL, total and ionized calcium, vitamin D, parathyroid hormone 1-84, bone alkaline phosphatase (BALP), osteocalcin, and N- and C-terminal telopeptides were assayed.

**Results:**

Overall, 60 women (mean age: 62.3 ± 7.5 years) received monthly IM NE for 6 years, with vitamin D and calcium supplementation in 81.3% of cases. Compared to BL, BMD increased significantly already after 1 year at all sites (4.5 ± 0.9% for L1-L4, 4.5 ± 0.8% for TF, and 2.1 ± 0.6% for FN, *P* ≤ 0.05), and the changes were maintained over time, whereas FN further improved up to year 3 and remained stable afterwards (*P* ≤ 0.05). All BMs, except for total calcium and BALP, progressively decreased over time (*P* ≤ 0.05). No fractures and significant adverse events were reported.

**Conclusion:**

The monthly administration of IM NE represents a manageable and effective option, in terms of BMD and bone BM improvement, for the long-term treatment of postmenopausal OP women with gastric intolerance to BPs. This trial is registered with ClinicalTrials.gov Identifier: NCT03699150.

## 1. Introduction

Osteoporosis (OP) is the most common metabolic bone disease [1], and it is characterized by the loss of bone mass and strength due to nutritional, metabolic, or pathological factors. Therefore, OP exposes the individual to an increased risk of fractures as a consequence of the reduced bone density and altered bone microarchitecture, finally resulting in poor quality and expectancy of life [2].

OP is increasing with the progressive aging of the population, affects a high proportion of women in postmenopause, and has high social-economic burden for the serious consequences that it entails [3]. The most common treatment for OP is represented by oral bisphosphonate (BPs). Nevertheless, the modality of BP administration makes their use rather uncomfortable for many patients and inaccessible to those compelled to bed. In fact, most BPs have to be taken orally under fasting conditions in the morning and patients are required to remain standing for at least half an hour after administration. In addition, the gastrointestinal (GI) tolerance of oral BPs is poor and the GI absorption is limited and variable. All these conditions result in poor compliance, that, in turn, leads to low efficacy and increased risk of fracture [4, 5].

Neridronate (6-amino-1-idroxyesilidene-1,1-bisphosphonate) (NE) is a nitrogen-containing BP licensed in Italy for the treatment of osteogenesis imperfecta and Paget's disease of bone, but it is effective also in other skeletal diseases such as OP, algodystrophy, hypercalcemia of malignancy, and bone metastases [6]. It has been developed for parenteral use only, and it can be administered intramuscularly (IM), thus avoiding all the limitations of oral BPs while easing home treatment.

For these reasons, and on the basis of scientific evidences [7–9], the Tuscany Region Health Committee extended the use of NE to OP patients with or without peptic ulcers (stomach and duodenal), hiatal hernia, gastroesophageal reflux disease, and resistance to other BPs (law GRT n. 836 20/10/2008) [10]. In pilot randomized controlled studies (RCTs) conducted in postmenopausal OP women, NE given intravenously (IV) yielded an increase in bone mineral density (BMD) at the lumbar spine (L1-L4) and femoral neck (FN), respectively [7–9]. Moreover, Cascella and coworkers demonstrated a statistically significant improvement of BMs after 3 months of treatment and of BMD after 12 months [8]. The efficacy of NE on BMD is dose-dependent for total hip but not for L1-L4, as demonstrated by Adami et al. [9]. Yet, in these trials, the entire study period ranged between 1 [8] and 3 years [7, 9], which is a limited time if compared to the time required to treat a chronic disease such as OP.

The aim of our study was to evaluate the long-term effects (over 6 years) of NE on BMD and circulating bone turnover marker (BM) levels in postmenopausal OP patients.

## 2. Methods

### 2.1. Study Design Population and Treatment

This is a single-center, prospective study including outpatients referred to the Gynecologic Endocrinology and Osteoporosis Unit of Fondazione CNR-Regione Toscana G. Monasterio, Pisa, Italy. Women were included if postmenopausal and diagnosed with OP based on BMD data, obtained at L1-L4, FN, and TF, and with contraindication, intolerance, or resistance to oral BP treatment due to GI problems (in accordance with the Tuscany Region's law GRT n. 836 20/10/2008) [10]. Exclusion criteria comprised limited motility conditions, bone prosthetic surgery, previous osteoporotic vertebral and hip fractures and chronic kidney disease, treatment with glucocorticoids, hormone replacement therapy, selective estrogen receptor modulators, psychotropic medication, anticonvulsants, and/or calcium. Moreover, women with the following conditions were excluded: gastrectomy, inflammatory bowel disease, malignant disease (i.e., of the stomach, esophagus, colon, lung, pancreas, liver, bile duct, gallbladder, breast, uterus, ovaries and bladder, or malignant lymphoma, leukemia, and multiple myeloma), type 1 diabetes mellitus, hyperthyroidism, hypo/hyperparathyroid disorder, rheumatoid arthritis, and/or other collagen diseases.

All patients received 25 mg IM NE once a month and, if necessary, supplementation with calcium (calcium carbonate) and vitamin D (colecalciferol) to reach circulating levels of calcium within the normal range and plasma concentrations of vitamin D (25-OH-D) ≥30 ng/mL. Due to the length of treatment with NE (i.e., 6 years), a placebo control group was not included for ethical reasons.

The study was approved by the Local Ethical Committee (Prot n° 37981, study 3605, 20/06/2012). All women gave written informed consent to their participation in the study, which was conducted in accordance with the Declaration of Helsinki. The Clinical Study Registration number at ClinicalTrials.gov was NCT03699150.

### 2.2. Clinical and Laboratory Assessments

BMD was assessed at L1-L4, FN, and TF through dual-energy X-ray absorptiometry (DEXA, Explorer QDR Series bone densitometer, Hologic, Marlborough, MA, USA) according to the manufacturer's instructions, which included a quality control test using a standard phantom. DEXA was performed at baseline (BL) and every 12 months until the study end. The ratio between the bone mineral content and area, in square centimeters, was expressed as the *T*-score, calculated as a standard deviation score from a normal reference population database [11]. Data were classified as follows: *T* − score≥−1 = normal, −1 > *T* − score>−2.5 = low bone density (osteopenia), and *T* − score≤−2.5 = OP [11].

Fasting blood sampling for the evaluation of bone metabolism biomarkers and the medical examination were performed between 7.00 and 9.30 AM at BL, month 3, and every 12 months after BL until the study end.

Specifically, blood samples were taken after an overnight fast and centrifuged at 2500 g, for 10 min. Then, samples, if not immediately assayed, were stored at –80°C until assayed, for the following biomarkers: total-calcium (t-Ca; heparinized plasma; Biochemistry, CX9 Chemistry Analyzer, Beckman, CA, USA), ionized calcium (i-Ca; external reference laboratory), serum vitamin D (25(OH)D; Liason, DiaSorin, Italy), bone alkaline phosphatase (BAP; Liason, DiaSorin, Italy), osteocalcin (OC; Liason, DiaSorin, Italy), C-terminal telopeptides of type I collagen (CTX; ECLIA Roche Diagnostics, Indianapolis, IN) and N-telopeptides of type I collagen (NTX; external reference laboratory), parathyroid hormone 1-84 (PTH; sample maintenance at 4°C, plasma EDTA, Liason DiaSorin, Italy), and homocysteine, folates, and B12 vitamin (samples maintenance at 4°C, serum, Architect, Abbott). The evaluation of other biochemical markers (e.g., urea, creatinine and lipid profile, and heparinized plasma) was performed by standard automated laboratory instruments.

Data about cardiovascular risk factors and comorbidities were also recorded: hypertension, dyslipidemia and hypercholesterolemia, type 2 diabetes, hypothyroidism [12], and hypo-vitamin B12 and/or hypofolatemia.

### 2.3. Statistical Analysis

Continuous variables were reported as mean ± standard deviation (SD) and range (minimum-maximum) and categorical variables as frequencies. Owing to skewness, the log transformation of some parameters (i.e., PTH, OC, BAP, and CTX) was performed. Log-transformed values were then back-transformed for data presentation. All the analyses were carried out by StatView, version 5.0.1 (SAS Institute, Abacus Concept Inc., Berkeley, CA, USA), and included *t*-test, simple regression analysis, and analysis of variance (ANOVA). Bonferroni correction was used to adjust for multiple comparisons in order to ensure an overall nominal significance level of 0.05. *P* values were two-tailed.

## 3. Results

### 3.1. Patient Characteristics

Between January 2008 and December 2016, 67 women were eligible for the study. 60 postmenopausal osteoporotic outpatients (mean age at study entry: 62.3 ± 7.5 years; mean age at menopause: 49.2 ± 5.5 years) accepted to participate and were included in the study. The baseline characteristics are listed in Table 1. Along the study period, 4 patients dropped out (1 patient at 18 months, 2 patients during the third year, and 1 patient at the fifth year of treatment). Briefly, 85% (51/60) of patients were nonsmokers, 13.3% (8/60) were ex-smokers, and 1.7% (1/60) was current smokers. The mean body mass index was 23.9 ± 4.1 kg/m^2^ (i.e., within the normal range). The most frequent comorbidities were dyslipidemia (52/60, 86.7%), hypercholesterolemia (48/60, 80%), hypertension (42/60, 70%), and hypothyroidism (30/60, 50%). Table 1 lists also the BL DEXA values: the mean BMD was 0.745 ± 0.085 g/cm^2^ for L1-L4, 0.601 ± 0.069 g/cm^2^ for FN, and 0.685 ± 0.096 g/cm^2^ for TF.

**Figure 1:**
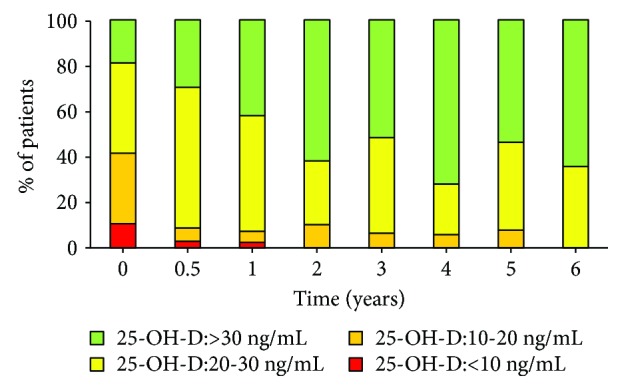
Patient distribution by the levels of circulating vitamin D (25-OH-D) as determined during the study period.

BL bone and biochemical parameters are reported in Table 2. At BL, only 18.7% (11/60) of patients had sufficient levels of 25-OH-D (>30 ng/mL) and, therefore, did not receive Ca and vitamin D supplementation.  Figure 1 shows the levels of 25-OH-D (<10 ng/mL, between 10 and 20 ng/mL, between 20 and 30 ng/mL, and > 30 ng/mL) across the patient population at each time point.

### 3.2. Bone Densitometry Assessment

**Figure 2:**
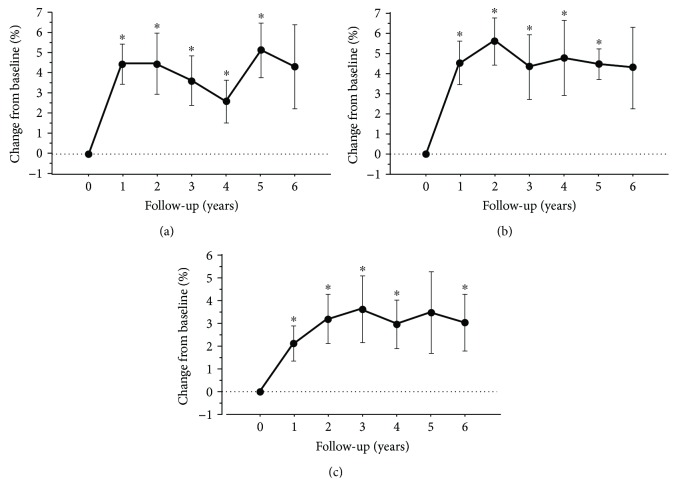
Mean change from baseline of BMD at L1-L4 (a), TF (b), and FN (c) over time. Data are presented as mean ± standard deviation. ^∗^*P* ≤ 0.05 vs baseline. Abbreviations: BMD: bone mineral density; L1-L4: lumbar spine; TF: total femur; FN: femoral neck.

The BMD changes over time at L1-L4, TF, and FN are shown in Figures 2(a) and 2(c). A statistically significant increase in BMD at all bone sites was observed already after 1 year of treatment (mean change from BL: 4.5 ± 0.9% for L1-L4, 4.5 ± 0.8% for TF, and 2.1 ± 0.6% for FN, *P* < 0.05). The improvement yielded by NE at L1-L4 and TF was maintained over time, from year 2 to 6 vs BL (*P* ≤ 0.05), whereas FN BMD progressively rose up to year 3 and remained stable thereafter.

### 3.3. Bone Turnover Biomarker Assessment

**Figure 3:**
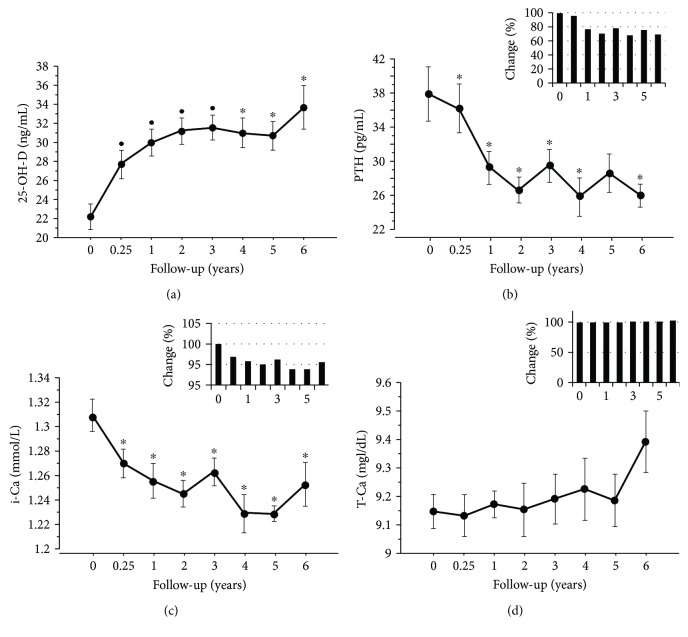
Levels of 25-OH-D (a), PTH (b), i-Ca (c), and t-Ca (d) over time. Data are presented as mean ± standard deviation. Histograms in panels (b-d) indicate the mean change from baseline (%) of each biomarker. ^∗^*P* ≤ 0.05 vs baseline, ^•^*P* ≤ 0.001 vs baseline. Abbreviations: 25-OH-D: vitamin D; PTH: parathyroid hormone; i-Ca: ionized calcium; t-Ca: total calcium.

**Figure 4:**
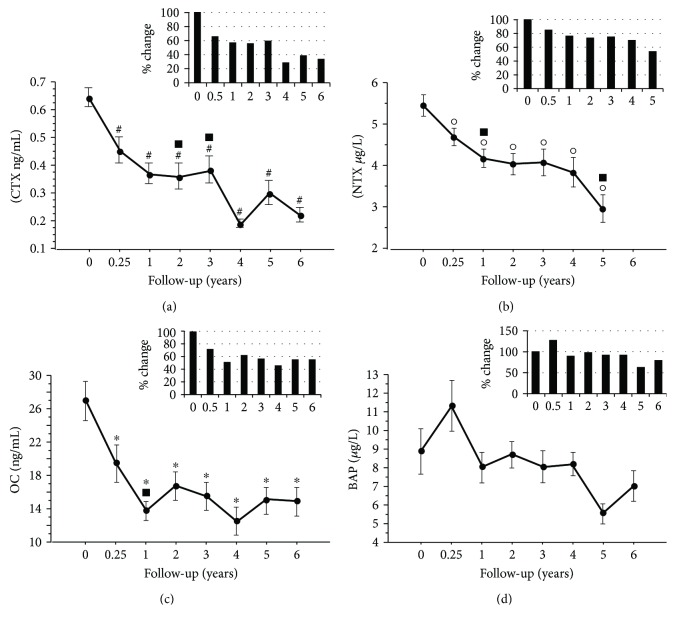
Levels of bone biomarkers over time: CTX (a), NTX (b), OC (c), and BAP (d). Data are presented as mean ± standard deviation. Histograms indicate the mean change from baseline (%) of each biomarker. ^∗^*P* ≤ 0.05 vs baseline, ^○^*P* ≤ 0.01 vs baseline, ^#^*P* ≤ 0.0001 vs baseline, ^▪^*P* ≤ 0.05 vs 0.25 years. Abbreviations: CTX: C-terminal telopeptide; NTX: N-terminal telopeptide; OC: osteocalcin; BALP: bone alkaline phosphatase.

Figures 3 and 4 the show change of biochemical parameters over time. A significant increase in 25-OH-D levels was recorded after 3 months of treatment compared to BL (*P* ≤ 0.001) which became optimal (≥30 ng/mL) at 1 year and remained stable afterwards (*P* ≤ 0.05) (Figure 3(a)).

The levels of PTH, i-Ca (Figures 3(b) and 3(c)), CTX, NTX, and OC (Figures 4(a) and 4(c)) significantly and progressively decreased compared to BL (*P* ≤ 0.05), even if they persisted within the normal range. No significant changes were observed in t-Ca levels (Figure 3(d)), whereas BAP tended to decrease (Figure 4(d)), but the difference vs BL did not reach significance at any time point.

A negative correlation was observed between the levels of 25-OH-D and those of CTX (*r* = −0.33, *P* ≤ 0.0001) and NTX (*r* = −0.31, *P* ≤ 0.0002).

Throughout the study period, no fractures and significant adverse events were reported. The dropout rate was 7%.

## 4. Conclusions

The findings presented here show that the 6-year monthly administration of 25 mg IM NE yielded a significant BMD increase at both spine and femur sites and an improvement of BMs in postmenopausal OP women, with no adverse events, fractures, or patient dropout. To our knowledge, the treatment period of this study is the longest reported so far with NE, and this is important considering that OP is a chronic disease that requires long-term therapies.

We observed a significant increase in BMD already after 1 year of treatment at L1-L4 and TF, which was maintained over time, while, at FN, it further improved up to year 3 and remained stable afterwards. These data are in line with the results from previous studies demonstrating the efficacy of NE in the treatment of OP [7–9]. However, these trials had limited treatment time (1-2 years) and employed different doses and routes of administration. In an early pilot RCT conducted in 78 postmenopausal OP women, NE was given IV at the dose of 50 mg once every 2 months over 2 years [7]. BMD increased by 6.2% and 3.7% at the L1-L4 and FN, respectively, in the first year and by 7.4% and 5.8% in the second year. During the first year after treatment discontinuation, spine BMD remained unchanged while hip BMD slightly decreased, but the differences versus BL remained highly significant [7]. Similar results were obtained in another pilot study conducted in 40 postmenopausal women treated with 25 mg IM NE given monthly for 1 year [8]. Moreover, a dose-finding clinical, multicenter trial included 188 postmenopausal OP women randomized to IM therapy with 25 mg NE every 2 weeks, 12.5 or 25 mg every 4 weeks, or placebo [9] for 12 months and followed up for 2 years post-treatment. All the three doses were associated with a significant BMD increase at both total hip and spine. A significant dose–response relationship for the different doses was observed for the BMD changes at the total hip but not at the spine.

In the TRIO study, the effects of oral alendronate, ibandronate, and risedronate on BMD were compared over 2 years in 172 postmenopausal women with OP. An increase in L1-L4 (4.1 ± 3.1%) and FN BMD (2.3 ± 3.9%) was observed during the first year, similarly to our findings. Nevertheless, our data showed a more evident BMD boost at the TF in comparison to oral BPs [13]. In the review of Eriksen et al. comparing the long-term effects of four bisphosphonates confirmed similar data [14]. Indeed, data of meta-analysis of Inderjeeth et al., studying the efficacy, safety, and adherence rates to both oral and intravenous ibandronate treatment, showed BMD changes similar to our findings: an improvement ranging between 3.4% and 4.9% [15].

The bone marker evaluation within the first 3-6 months of treatment is important to assess the effects of antiresorptive BP therapies and optimize therapy adherence [16]. In the present study, an improvement in the levels of BMs was observed by the third month of treatment and was maintained up to the study end. These results are consistent with previous data regarding the effects of both oral BPs and NE [8, 17]. In our study, the levels of CTX decreased by 61% and 66% at years 5 and 6, respectively, and NTX by 46% at the end of the study. The literature reports also a latter decrease in bone formation markers of approximately 30-40%, which is comparable to our results of OC trend over time (Figure 4(c)) [17–20].

Similar to the general population [21, 22], most of patients had insufficient 25-OH-D circulating levels. Many studies confirmed the ineffectiveness of BPs (or antiresorptive treatments) related to when the levels of 25-OH-D are low [23]. In particular, Carmel and coworkers demonstrated the importance of restoring vitamin D levels within the normal range before starting treatment, to improve and optimize the efficacy of BPs by increasing intestinal calcium absorption, bone mineralization, and renal reabsorption of calcium and phosphate ions [24]. Therefore, in the present study, vitamin D supplementation was provided to those women with 25-OH-D levels <30 ng/mL (i.e., 81.3%). Notably, 25-OH-D levels significantly increased after 3 months of treatment, were optimal at year 1 (≥30 ng/mL) and remained stable thereafter.

We observed a significant inverse correlation between 25-OH-D, CTX, and NTX levels, indicating a major decline on bone turnover rate in patients with proper vitamin D therapy management. von Hurst and colleagues showed increased CTX levels (*P* = 0.001) in women >49 years or postmenopausal who were not vitamin D-supplemented, supporting an increased rate of bone turnover. In the same population, however, vitamin D supplementation correlated with CTX decrease (*P* = 0.012) [25, 26].

Long-term adherence to treatment of a relatively asymptomatic chronic disease is usually scarce, and OP is not an exception. In fact, compliance to oral BP treatment has been reported to be approximately 50% at 6 months [27] and <40% after 1 year [28], and this is important in light of the fact that antifracture efficacy is linked to treatment persistence [29, 30]. In the present study, only 7% of patients treated dropped out along the 6 years of study, suggesting that the single monthly administration of IM NE and the absence of side effects favor the adherence to long-term therapy.

The present study has some limitations. First of all is the lack of a placebo group. However, in light of the results from previous RCTs in which the control arm received calcium and vitamin D supplementation and had no benefit in terms of BMD and bone turnover markers [28], we judged as no ethical to provide supplementation alone for 6 years to subjects at high risk of fractures. Moreover, NE could be administered only to OP patients with gastric intolerance to BPs. Yet, this restriction depends on the Tuscany Region's law GRT n. 836 2008, which permits the off-label administration of NE to this selected population only.

BP use for more than 5 years seems to be associated with an increased relative risk of atypical femur fractures (AFF), although the absolute risk is low (3.2–100 cases per 100,000 person-years). Longer therapies enhance the risk of AFF [31–33]. At the same time, the benefit on the typical hip fracture reduction generally outweighs the risk of AFF, especially in high-risk individuals. In our study, the patient mean age was 62.3 ± 7.5 years at BL and none of them experienced typical and/or atypical fractures throughout the 6 years of treatment.

In conclusion, our findings support the long-term use of monthly IM NE to treat OP in postmenopausal women with gastric intolerance to oral BPs. The ease of use of this formulation, which favors home administration, along with the benefit in terms of BMD and bone turnover and the lack of fractures and adverse events contribute to the increase in therapy compliance. This is crucial in the real-world setting of a chronic disease such as OP, which requires long-term treatment.

## Figures and Tables

**Table 1:** Patient characteristics and DEXA parameters at baseline.

Characteristics	Overall population (*N* = 60)
Age at study entry (years)	62.3 ± 7.5 (49.5-80.4)
Age at menopause (years)	49.2 ± 5.5 (35.0-55.0)
Smoking status	
Never	51 (85)
Former	8 (13.3)
Current	1 (1.7)
BMI (kg/m^2^)	23.9 ± 4.1 (18.8-31.9)
SBP (mmHg)	131 ± 22 (100-172)
DBP (mmHg)	73 ± 10 (55-88)
HR	72 ± 9 (50-89)
Comorbidities	
Dyslipidemia	52 (86.7)
Hypercolesterolemia	48 (80)
Hypertension	42 (70)
Hypothyroidism	30 (50)
Hypo-vitB12 or folate	6 (10)
DM2	2 (3.3)
DEXA parameter	
*T*-score L1-L4	−2.7 ± 0.8 (−4.2-0.8)
*T*-score FN	−2.2 ± 0.6 (−3.3-0.6)
*T*-score TF	−2.2 ± 0.7 (−3.4-0.7)
BMD (g/cm^2^) L1-L4	0.745 ± 0.085 (0.542-0.935)
BMD (g/cm^2^) FN	0.601 ± 0.069 (0.485-0.739)
BMD (g/cm^2^) TF	0.685 ± 0.096 (0.530-0.923)

Data are expressed as mean ± standard deviation (SD) and range (min-max), or frequencies (*N* [%]). Abbreviations: BMI: body mass index; SBP: systolic blood pressure; DBP: diastolic blood pressure; HR: heart rate; vitB12: vitamin B12; DM2: diabetes mellitus type 2; L1-L4: lumbar spine; FN: femoral neck; TF: total femur; BMD: bone mineral density.

**Table 2:** General and bone biochemical parameters at baseline.

Parameter	Overall population (*N* = 60)	Reference values
*General biochemical parameters*		
Uric acid (mg/dL)	4.6 ± 0.8 (3.4-6.6)	<6
Urea (mg/dL)	33.6 ± 8.2 (17.4-48.9)	12.6-42.6
Creatinine (mg/dL)	0.75 ± 0.14 (0.46-1.06)	<1.30
Total protein (g/dL)	7.16 ± 0.41 (5.77-7.84)	6.00-8.20
Fasting plasma glucose (mol/L)	4.94 ± 0.50 (4.00-5.78)	3.3-6.1
Glycosylated Hb (%)	5.8 ± 0.4 (5.2-6.8)	4.0-6.0
Total cholesterol (mg/dL)	210 ± 29 (160-309)	120-200
HDL (mg/dL)	64 ± 15 (41-100)	≥45
LDL (mg/dL)	126 ± 24 (79-172)	≤160
Triglycerides (mg/dL)	92 ± 42 (35-214)	30-150
Vitamin B12 (pg/mL)	402 ± 190 (198-803)	187-883
Folates (ng/mL)	7.6 ± 2.4 (3.9-13.3)	3.1-20.5
Homocysteine (*μ*mol/L)	12.7 ± 4.4 (7.6-25.0)	4.4-13.6
*Bone biochemical parameters*		
25(OH)D (ng/mL)	22.9 ± 9.4 (4.0-45.7)	<10.0 insufficiency, 10.0-30.0 deficiency 30.0-100.0 normality
PTH (pg/mL)	37.9 ± 21.6 (11.8-98.5)	4.4-58.6
T-Ca (mmol/L)	2.25 ± 0.10 (2.47-2.02)	2.10-2.62
I-Ca (mmol/L)	1.31 ± 0.09 (1.04-1.40)	1.13-1.32
CTX (ng/mL)	0.64 ± 0.23 (0.058-0.956)	<0.779
NTX (*μ*g/L)	5.5 ± 1.7 (1.7-12.2)	2.1-5.6
Osteocalcin (ng/mL)	29.9 ± 14.7 (3.56-59.2)	5.0-60.0
BAP (*μ*g/L)	10.9 ± 8.9 (2.7-39.0)	6.0-26.0

Data are expressed as mean ± standard deviation (SD) and range (min-max). Abbreviations: Hb: hemoglobin; HDL: high-density lipoprotein; LDL: low-density lipoprotein; 25-OH-D: circulating vitamin D; PTH: parathyroid hormone, T-Ca: total calcium; I-Ca: ionized calcium; CTX: carboxy-terminal cross-linked telopeptide of type 1 collagen; NTX: amino-terminal cross-linked telopeptide of type 1 collagen; BAP: bone alkaline phosphatase.

## Data Availability

The data used to support the findings of this study are available from the corresponding author upon request.

## Abbreviations

BL: Baseline
BPs: Bisphosphonates
BALP: Bone alkaline phosphatase
BMD: Bone mineral density
BMs: Bone turnover biomarkers
DEXA: Dual-energy X-ray absorptiometry
CTX: C-terminal telopeptide
FN: Femoral neck
GI: Gastrointestinal
i-Ca: Ionized calcium
IM: Intramuscular
L1-L4: Lumbar spine
NE: Neridronate
NTX: N-terminal telopeptide
OC: Osteocalcin
OP: Osteoporosis
PTH: Parathyroid hormone 1-84
t-Ca: Total-calcium
TF: Total femur.
